# Effects of Isokinetic Knee Extension Explosive Strength on Maximum Walking Speed in Patients With Knee Osteoarthritis

**DOI:** 10.7759/cureus.97731

**Published:** 2025-11-25

**Authors:** Bungo Ebihara, Hayato Miyasaka, Koichi Iwai, Takashi Fukaya, Shigeki Kubota, Makoto Takahashi, Hirotaka Mutsuzaki

**Affiliations:** 1 Department of Rehabilitation, Tsuchiura Kyodo General Hospital, Tsuchiura, JPN; 2 Graduate School of Health Sciences, Ibaraki Prefectural University of Health Sciences, Ami, JPN; 3 Centre for Humanities and Sciences, Ibaraki Prefectural University of Health Sciences, Ami, JPN; 4 Department of Physical Therapy, Faculty of Health Sciences, Tsukuba International University, Tsuchiura, JPN; 5 Department of Occupational Therapy, Faculty of Health and Medical Sciences, Ibaraki Prefectural University of Health Sciences, Ami, JPN; 6 Department of Physical Therapy, School of Health Sciences, Japan University of Health Sciences, Satte, JPN; 7 Centre for Medical Science, Ibaraki Prefectural University of Health Sciences, Ami, JPN; 8 Department of Orthopaedic Surgery, Ibaraki Prefectural University of Health Sciences, Ami, JPN

**Keywords:** explosive strength, isokinetic, knee osteoarthritis, peak torque, walking speed

## Abstract

Objective

This study aimed to identify the effects of isokinetic knee extension explosive strength on maximum walking speed in individuals with knee osteoarthritis (OA).

Methods

This study included 30 participants with knee OA (mean age: 74.9 ± 6.9 years). An isokinetic dynamometer was used to measure knee extension torque at an angular velocity of 60°/s. Walking speed was calculated using the 10-m walking test. Participants were allowed to use a cane if they normally used one. Measured variables included explosive strength at 0.02, 0.1, 0.2, and 0.3 s after the onset of knee extension; knee extension peak torque; knee range of motion; knee pain; and maximum walking speed. A propensity score for cane use during the 10-m walking test was calculated using logistic regression analysis. Generalized linear models were then used to analyze the effect of knee extension strength on maximum walking speed, applying inverse probability weighting using the propensity score.

Results

The generalized linear model with the explosive strength at 0.02 s after the start of knee extension showed the highest goodness of fit (Akaike information criterion = 10.663, Schwarz’s Bayesian information criterion = 16.953). The explanatory variables for maximum walking speed were the explosive strength at 0.02 s after the start of knee extension, age, and whether or not a cane was used (p = 0.015, 0.012, 0.036, respectively).

Conclusion

Isokinetic knee extension explosive strength had a greater influence on maximum walking speed than peak torque. Our findings suggest that training to improve isokinetic explosive strength could enhance walking speed.

## Introduction

Knee osteoarthritis (OA) is a chronic condition that leads to progressive structural deterioration and functional impairment of the knee joint [1]. As the severity of knee OA progresses, gait function, activities of daily living, and quality of life decline [2,3]. The prevalence of knee OA among individuals aged ≥60 years is estimated to be 47.0% in men and 70.2% in women [4]. Preventing disease progression is, therefore, a key public health issue [5]. The maximum walking speed can be used to predict a decline in activities of daily living [6]. Thus, reduced walking speed in individuals with knee OA has important public health implications.

Knee joint functions related to walking speed include muscle strength, range of motion (ROM), and pain [7-9]. Knee extension strength is positively correlated with walking speed [10,11]. Notably, isometric explosive strength can be associated with maximum walking speed [12,13]. Isometric explosive strength can be calculated as the slope of the torque-time curve or force-time curve during a short time interval after the onset of muscle contraction [12,14]. It is involved in daily activities that require rapid muscle contractions [14]. In severe knee OA, which leads to a decline in activities of daily living, isometric explosive strength is reduced [15]. The knee extension explosive strength is a key muscle function in patients with knee OA.

Isokinetic muscle testing is the gold standard for assessing dynamic muscle strength and can detect strength changes as knee OA progresses [16,17]. Furthermore, isokinetic muscle strengthening is more effective than isotonic or isometric muscle strengthening for knee OA [18]. Isokinetic motor function is important. However, little is known about the effect of isokinetic knee extension explosive strength on maximum walking speed.

Therefore, in this study, we aimed to identify the effects of knee extension explosive strength on maximum walking speed in individuals with knee OA using isokinetic testing. Our hypothesis was that isokinetic explosive strength would have a greater influence on maximum walking speed than peak torque.

## Materials and methods

Study design

This study was conducted among individuals with knee OA between June 2022 and September 2023. Data on knee joint function were collected retrospectively. The study was approved by the Ethics Committee of Tsuchiura Kyodo General Hospital (Approval No. 2024FY95) and conducted in accordance with the tenets of the Declaration of Helsinki. Participant consent was obtained through an opt-out process.

Participants

Thirty individuals with knee OA admitted for total or unicompartmental knee arthroplasty were enrolled. The inclusion criterion was the ability to walk independently with or without a cane. Individuals who required rest or assistance during the 10-m walking test and who could not understand how to perform muscle strength tests due to cognitive decline were excluded. The affected limb was used for measurement. For bilateral OA, the limb with the greater reduction in knee extension peak torque was selected. Data on age, sex, height, weight, and Kellgren-Lawrence (KL) grade were extracted from medical records [19].

Knee extension strength

**Figure 1:**
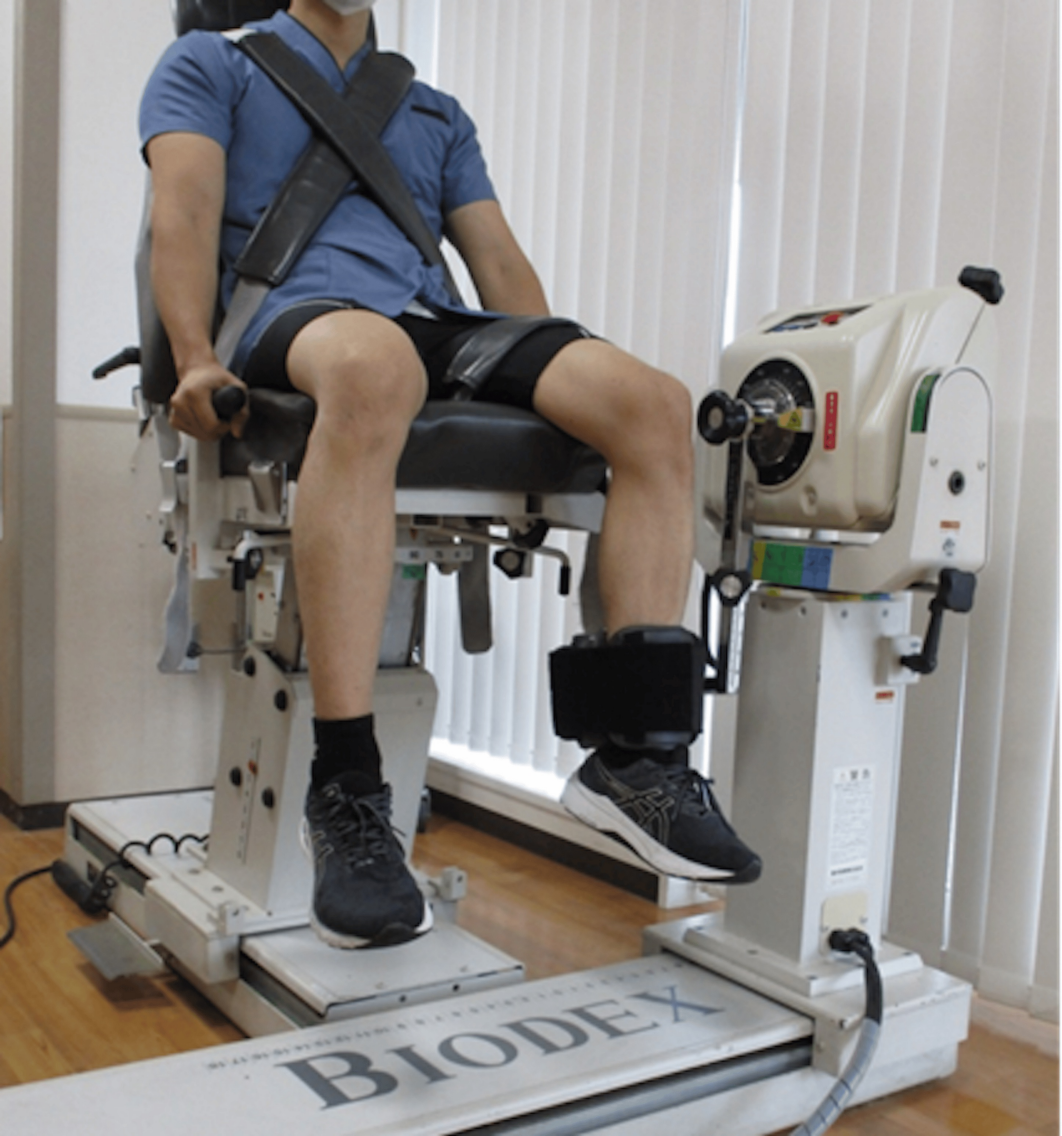
Measurement of knee extension strength Knee extension strength was measured using an isokinetic dynamometer. The participant’s trunk and thigh of the test limb were secured to the backrest and seat with belts. The dynamometer was fixed to the distal portion of the lower leg, also using a belt.

Knee extension strength was assessed using an isokinetic dynamometer (Biodex System 3C dynamometer, Biodex Medical Systems, Inc., Shirley, NY). Participants sat on the dynamometer seat. The examiner secured the trunk and thigh of the test limb to the backrest and seat with belts, and the dynamometer was fixed to the distal lower leg, also using a belt (Figure 1). Gravity correction was applied. Participants were instructed to extend the knee with maximum effort. Through explanations and practice, the participants were able to fully understand the exercise method. Each participant completed two sets of five isokinetic knee extensions at an angular velocity of 60°/s, with verbal encouragement. The angular velocity of 60°/s is most commonly used in isokinetic muscle strength testing [20]. A 25-s rest was administered between sets.

Data were extracted from the set that produced the highest peak torque. Recorded variables included peak torque, time to peak torque, knee ROM during the test, and torque values at 0.02, 0.1, 0.2, and 0.3 s after the onset of knee extension. All torque values were normalized to body weight. Additionally, the explosive strength was calculated by dividing the torque values at 0.02, 0.1, 0.2, and 0.3 s after the onset of knee extension by each time.

Knee ROM

Knee ROM was measured using a goniometer. Participants lay in a supine position on a bed, and the measurer passively moved the knee through its full range. The angle between the thigh and lower leg axes was recorded as the extension and flexion ROM. The thigh axis was defined by the line from the greater trochanter to the lateral femoral condyle, and the lower leg axis by the line from the fibular head to the lateral malleolus.

Knee pain during walking

Knee pain during level-ground walking was assessed using a numerical rating scale. A measurement sheet displayed a 0-10 scale in 1-point increments, with 0 indicating no pain and 10 indicating maximum pain. Participants circled the number that best described their pain.

Maximum walking speed

The time to complete the 10-m walk was recorded using a stopwatch. A straight, level 10-m path was marked with red tape at the start and finish lines. Participants were instructed to walk as fast as possible. Those who normally used a cane were permitted to do so. Walking began 2 m before the start line and continued beyond the finish line [21]. The examiner, walking alongside, started and stopped the stopwatch when the lower limb crossed each tape line. The maximum walking speed was calculated from the walking time. The 10-m walk test is reliable and easy to administer in clinical settings [21]. There is an excellent correlation between the results of this test and the walk distance in the six-minute walk test, a common performance assessment in knee OA [22].

Statistical analysis

Descriptive statistics were computed. The Shapiro-Wilk test assessed data normality. Normally distributed variables were presented as means and standard deviations; otherwise, medians and interquartile ranges were reported.

Correlation analyses were conducted to investigate the relationship between knee extension strength and walking speed. Pearson’s product-moment correlation coefficients were used for height, weight, body mass index, knee extension ROM, flexion ROM, and peak torque. Spearman’s rank correlation coefficients were applied for age and explosive strength values at 0.02, 0.1, 0.2, and 0.3 s. Polyserial correlation coefficients were used for sex, KL grade, cane use during the 10-m walk, and knee pain during walking. 

A propensity score analysis was conducted for cane use during the 10-m walk as a potential confounder. Logistic regression estimated the predicted probability using knee pain and knee extension ROM as explanatory variables. Model fit was assessed using the Nagelkerke R², Cox and Snell R², and c-statistic. Generalized linear models examined the effects of knee extension strength, age, and cane use on walking speed, with inverse probability weighting based on propensity scores. Model fit was evaluated using the Akaike information criterion (AIC) and Schwarz’s Bayesian information criterion (BIC). 

SAS version 6 (SAS Institute, Cary, NC) was used to compute polyserial correlations; all other analyses were conducted using SPSS Statistics version 30 (IBM Corp., Armonk, NY). A p-value < 0.05 was considered statistically significant.

## Results

Physical characteristics

Data from 30 participants (13 men and 17 women) were collected. The physical characteristics are summarized in Table 1. None of the participants met the exclusion criteria. The mean age was 74.9 ± 6.9 years. The KL grade was 2 in three participants, 3 in nine participants, and 4 in 18 participants.

**Table 1:** Physical characteristics BMI, body mass index; KL grade, Kellgren-Lawrence grade

Physical characteristics	n = 30
Age (years)	74.9 ± 6.9
Men	13
Women	17
Height (m)	1.58 ± 0.09
Weight (kg)	63.5 ± 11.4
BMI (kg/m^2^)	25.4 ± 3.8
KL grade 1	0
KL grade 2	3
KL grade 3	9
KL grade 4	18

Measured values 

Measured values are summarized in Table 2. The mean maximum walking speed was 1.14 ± 0.39 m/s, and the mean knee extension peak torque was 0.74 ± 0.31 Nm/kg. The explosive strength at 0.02 s after the start of extension was 8.17 (5.77 to 15.34) Nm/kg/s. Five participants used a cane during the 10-m walking test. Pain during walking was not reported by four participants.

**Table 2:** Measured values ROM, range of motion; Explosive strength 0.02, explosive strength at 0.02 seconds after the start of extension; Explosive strength 0.1, explosive strength at 0.1 seconds after the start of extension; Explosive strength 0.2, explosive strength at 0.2 seconds after the start of extension; Explosive strength 0.3, explosive strength at 0.3 seconds after the start of extension

Measurement items		Values
ROM	Extension (degrees)	-10.0 (-15.0 to -5.0)
	Flexion (degrees)	125.5 ± 18.1
Extension strength	Peak torque (Nm/kg)	0.74 ± 0.31
	Time to reach peak torque (s)	0.59 ± 0.21
	Peak torque angle (degrees)	71.6 ± 11.5
	Explosive strength 0.02 (Nm/kg/s)	8.17 (5.77 to 15.34)
	Explosive strength 0.1 (Nm/kg/s)	2.40 (1.44 to 4.39)
	Explosive strength 0.2 (Nm/kg/s)	1.90 (1.35 to 3.19)
	Explosive strength 0.3 (Nm/kg/s)	1.50 (1.21 to 2.39)
	ROM during strength test (degrees)	90.1 ± 11.7
Walking	Pain (points)	6.7 ± 2.3
	Maximum walking speed (m/s)	1.14 ± 0.39

Correlation analysis

The results of the correlation analysis are summarized in Table 3. Maximum walking speed exhibited significant positive correlations with sex (p = 0.002); height (p = 0.012); knee extension peak torque (p = 0.003); and explosive strength at 0.02 s (p = 0.002), 0.1 s (p < 0.001), 0.2 s (p < 0.001), and 0.3 s (p < 0.001) after the start of extension. Maximum walking speed exhibited significant negative correlations with age (p = 0.038) and cane use (p < 0.001).

**Table 3:** Correlation analysis ROM, range of motion; BMI, body mass index; KL grade, Kellgren–Lawrence grade; Explosive strength 0.02, explosive strength at 0.02 seconds after the start of extension; Explosive strength 0.1, explosive strength at 0.1 seconds after the start of extension; Explosive strength 0.2, explosive strength at 0.2 seconds after the start of extension; Explosive strength 0.3, explosive strength at 0.3 seconds after the start of extension; Cane, cane use during the 10-m walking test; ᵃPearson’s product–moment correlation coefficient; ᵇSpearman’s rank correlation coefficient; ᶜPolyserial correlation coefficient; *Statistically significant

		Maximum walking speed	
		Correlation coefficient	p
Characteristics	Age	-0.381^b^	0.038^*^
	Sex	0.528^c^	0.002^*^
	Height	0.452^a^	0.012^*^
	Weight	0.326^a^	0.078
	BMI	0.036^a^	0.849
	KL grade	-0.022^c^	0.921
ROM	Extension	0.324^a^	0.081
	Flexion	0.122^a^	0.520
Extension strength	Peak torque	0.518^a^	0.003^*^
	Explosive strength 0.02	0.551^b^	0.002^*^
	Explosive strength 0.1	0.598^b^	<0.001^*^
	Explosive strength 0.2	0.582^b^	<0.001^*^
	Explosive strength 0.3	0.611^b^	<0.001^*^
Walking	Pain	-0.219^c^	0.261
	Cane	-0.766^c^	<0.001^*^

Propensity score analysis

Logistic regression analysis was used to calculate the predicted probability of cane use during the 10-m walking test and to generate the propensity score. The Nagelkerke R² was 0.485, the Cox & Snell R² was 0.303, the p-value for the Hosmer-Lemeshow test was p = 0.738, and the c-statistic was 0.900.

Generalized linear model

The generalized linear model results are summarized in Table 4. Model 1 used knee extension peak torque, age, and cane use as explanatory variables. In other models, explosive strength at 0.02-0.3 s after the start of knee extension was used in place of peak torque. In all models, knee extension strength, age, and cane use had significant effects (p < 0.05). The main model showed the best fit (AIC = 10.663, BIC = 16.953). Its explanatory variables were explosive strength at 0.02 s (EXP(B) = 1.02, p = 0.015), age (EXP(B) = 0.98, p = 0.012), and cane use (EXP(B) = 0.80, p = 0.036).

**Table 4:** Generalized linear model Explosive strength 0.02, explosive strength at 0.02 seconds after the start of extension; Explosive strength 0.1, explosive strength at 0.1 seconds after the start of extension; Explosive strength 0.2, explosive strength at 0.2 seconds after the start of extension; Explosive strength 0.3, explosive strength at 0.3 seconds after the start of extension; Cane, cane use during the 10-m walking test; AIC, Akaike information criterion; BIC, Schwarz’s Bayesian information criterion; *Statistically significant

	Main model		Model 1		Model 2		Model 3		Model 4	
	EXP(B)	p	EXP(B)	p	EXP(B)	p	EXP(B)	p	EXP(B)	p
Intercept	9.33	<0.001^*^	8.33	<0.001^*^	9.56	<0.001^*^	9.85	<0.001^*^	9.63	<0.001^*^
Peak torque	-	-	1.52	0.023^*^	-	-	-	-	-	-
Explosive strength 0.02	1.02	0.015^*^	-	-	-	-	-	-	-	-
Explosive strength 0.1	-	-	-	-	1.06	0.026^*^	-	-	-	-
Explosive strength 0.2	-	-	-	-	-	-	1.09	0.042^*^	-	-
Explosive strength 0.3	-	-	-	-	-	-	-	-	1.13	0.027^*^
Age	0.98	0.012^*^	0.98	0.017^*^	0.98	0.015^*^	0.98	0.013^*^	0.98	0.011^*^
Cane	0.80	0.036^*^	0.75	0.004^*^	0.77	0.012^*^	0.75	0.003^*^	0.74	0.002^*^
AIC	10.663		11.250		11.427		12.124		11.483	
BIC	16.953		17.541		17.718		18.414		17.774	

Overall, knee extension explosive strength, particularly explosive strength measured at 0.02 s, along with age and cane use, emerged as significant factors influencing maximum walking speed in individuals with knee OA.

## Discussion

In this study, we investigated the relationship between isokinetic knee extension explosive strength and maximum walking speed in individuals with knee OA. Explosive strength at 0.02 s after the start of knee extension had the greatest impact, supporting the hypothesis that explosive strength has a greater influence on walking speed than peak torque. Isokinetic explosive strength has potential clinical relevance for gait rehabilitation in knee OA. 

Our models identified explosive strength at 0.02 s as a stronger predictor of maximum walking speed than peak torque (AIC = 10.663 and BIC = 16.953 for the explosive strength model; AIC = 11.250 and BIC = 17.541 for the peak torque model). To the best of our knowledge, this is the first study to demonstrate the effect of explosive strength at 0.02 s assessed using isokinetic muscle testing on walking speed. During the early stance in normal gait, the ground reaction force initially passes anterior to the knee and shifts posteriorly by 0.02 s [23]. This transition increases both the external knee flexion moment and knee flexion angle [24]. Knee extensors contract rapidly to control this motion [23]. Therefore, explosive knee extensor activation during this phase is functionally critical for walking speed. By contrast, peak torque occurred at approximately 0.59 ± 0.21 s after extension initiation in this study, potentially too late to have an influence on early stance dynamics.

Our findings suggest that training to improve isokinetic explosive strength could enhance walking speed. High-velocity resistance training, which differs from high-intensity loading, may be better tolerated by individuals with knee OA [25]. High-intensity training can aggravate joint symptoms, whereas high-velocity resistance training has been shown to improve walking performance in older adults [26,27]. Thus, it may serve as an effective intervention for improving maximum walking speed in this population.

This study has some limitations. First, torque was measured only at an angular velocity of 60°/s, and the effects of other velocities remain unclear. However, the influence of the explosive strength at the most common angular velocity on maximum walking speed was identified. Second, only one knee joint was analyzed. Including other joints may yield models with higher explanatory power. Third, no three-dimensional motion analysis was performed. Fourth, this was a cross-sectional study with a small sample size and no control group or randomization process. These limitations should be considered when interpreting the generalizability of the findings, and they highlight the need for future studies using kinematic and kinetic approaches to clarify the biomechanical role of isokinetic explosive strength during walking.

## Conclusions

Explosive strength at 0.02 s after the start of knee extension had a greater effect on maximum walking speed in individuals with knee OA than did peak torque. These results suggest that isokinetic knee extension explosive strength plays a key role in walking speed. Our findings suggest that training to improve isokinetic explosive strength could enhance walking speed. Further research is needed to determine whether similar associations are true across different testing conditions and broader populations.
